# Incidence of *KRAS* G12C mutations in genitourinary malignancies; emerging target in precision medicine

**DOI:** 10.18632/oncotarget.28912

**Published:** 2026-08-19

**Authors:** Kelly Crane, K. R. Seetharam Bhat, P. Grivas, Andrea Necchi, Gennady Bratslavsky, Oleg Shapiro, Joseph M. Jacob, Hanan Goldberg, Rebecca Sager, Jeffery S. Ross

**Affiliations:** ^1^Department of Urology, SUNY Upstate Medical University, Syracuse, NY 13210, USA; ^2^Hospital San Raffaele, Milan, Italy; ^3^Foundation Medicine, Boston, MA 02210, USA; ^4^Upstate Urology at MVHS, Utica, NY 13502, USA

**Keywords:** KRAS mutation, genitourinary malignancy, precision medicine, emerging target

## Abstract

Purpose: The discovery of KRAS G12C as a therapeutic target has transformed oncology with the development of specific inhibitors. This study aimed to characterize the prevalence and clinicogenomic features of KRAS G12C mutations in genitourinary malignancies, an area with limited prior data despite recent FDA approvals of KRAS G12C-targeted therapies.

Materials and Methods: Comprehensive genomic profiling was performed on 13,654 formalin-fixed paraffin-embedded specimens, including 1,453 renal clear cell carcinomas (RCCC), 3,879 urothelial bladder carcinomas (UBC), and 8,322 prostate acinar adenocarcinomas (PAAC). Tumor mutational burden (TMB), microsatellite instability (MSI), and PD-L1 expression (Dako 22C3) were also assessed.

Results: KRAS alterations were identified in 7 RCCC (<1%), 202 UBC (5.2%), and 158 PAAC (1.9%) cases. KRAS G12C variants were detected in 0 RCCC (0%), 24 UBC (12%), and 1 PAAC (<1%) cases. No KRAS G12C-mutated tumors showed MSI-high status. In UBC, KRAS G12C-positive cases demonstrated significantly lower TERT co-mutations and higher KDM6A alterations compared to KRAS G12C-negative cases (both *p* < 0.05). Age and sex distributions were similar across subtypes. Predominant co-alterations included VHL, SETD2, and PBRM1 in RCCC, and TP53, CDKN2A, and TERT in UBC.

Conclusions: KRAS G12C mutations occur infrequently in major genitourinary malignancies. However, given the expanding therapeutic landscape of KRAS G12C inhibitors and potential for basket trials, further investigation in bladder and prostate cancers is warranted. These agents may offer meaningful benefit to the small subset of patients harboring this actionable alteration.

## INTRODUCTION

The KRAS oncogene ranks among the most well-characterized oncogenic drivers in human cancer biology. The G12C amino acid substitution variant specifically constitutes 12–15% of all KRAS-mutant malignancies and has recently emerged as a therapeutically tractable target with the development of novel pharmacologic agents [1, 2]. Sotorasib (Lumakras) achieved historic significance as the first United States Food and Drug Administration (FDA)-approved agent specifically targeting KRAS G12C alterations in non-small cell lung cancer (NSCLC) in May 2021, subsequently receiving commercial distribution authorization [2, 3]. The FDA has since granted approval to additional agents, with multiple active clinical trials currently investigating KRAS G12C inhibitors. This advancement in therapeutic targeting of malignancies harboring KRAS G12C alterations underscores the importance of evaluating inhibitor utility across tumors of varying histologic source [3].

Multiple papers have characterized the prevalence of KRAS G12C alterations across diverse cancer types. Data synthesized from COSMIC and American Cancer Society (ACS) 2018 cancer incidence statistics demonstrate that KRAS G12C occurs most frequently in lung adenocarcinoma (32.8%), colorectal adenocarcinoma (CRC) at 18.8%, and pancreatic adenocarcinoma at 4.6% [3, 4]. Lee and colleagues conducted a pan-cancer evaluation of KRAS-altered specimens, analyzing alterations prevalence in 24 distinct cancer types. Their findings largely corroborated earlier studies, identifying KRAS G12C alterations in NSCLC (14%), colorectal adenocarcinoma (CRC) at 3.5%, pancreatic ductal adenocarcinoma at 1.5%, bladder malignancies (<1%), and prostate adenocarcinoma (<1%) [2, 5, 6]. The present study was therefore designed to conduct a detailed analysis of KRAS G12C genomic alteration (GA) prevalence specifically within genitourinary malignancies, representing a relatively unexplored area despite substantial clinical importance following FDA authorization of multiple KRAS G12C inhibitors.

## RESULTS

A total of 13,654 specimens from adult patients with genitourinary malignancies were studied (Table 1). Overall, KRAS genomic alterations were identified in 367 (2.7%) specimens, with the KRAS G12C variant detected in 25 (0.2%) specimens. KRAS genomic alterations were identified in 7 RCCC (<1%), 202 UBC (5.2%), and 158 PAAC (1.9%). The G12C variant of KRAS was specifically identified in 0 RCCC (0%), 24 (12%) UBC, and 1 (<1%) PAAC of overall KRAS genomic alterations. Age and sex distributions demonstrated similarity across all 3 malignancy subtypes. No KRAS G12C-mutated tumors demonstrated MSI-high status.

**Table 1:** Distribution of KRAS mutations in genitourinary malignancies (*N* = 13,654)

	RCCC		UBC						PAAC					
	KRAS All mut		KRAS only G12C mut		KRAS no G12C mut		KRAS All mut		KRAS only G12C mut		KRAS no G12C mut		KRAS All mut	
Total Cases	7		24 (12%)		178 (88%)		202		1 (<1%)		157 (>99%)		158	
Age (median + range)	64 (52–70)		69 (55–84)		68 (37–89+)		68 (37–89+)		66 (N/A)		70 (38–89+)		70 (38–89+)	
Gender (M/F)	86%/14%		88%/12%		70%/30%		72%/28%		100%		100%/0%		100%/0%	
Other Gene Frequencies	*VHL*	86%	*TP53*	50%	*TERT*	68%	*TERT*	65%	STK11	100%	*TP53*	47%	*TP53*	47%
	*SETD2*	71%	*CDKN2A*	46%	*TP53*	47%	*TP53*	48%	APC	100%	*AR*	22%	*AR*	22%
	*PBRM1*	29%	*TERT*	46%	*CDKN2A*	40%	*CDKN2A*	41%			*PTEN*	18%	*PTEN*	18%
	*ARID1A*	14%	*CDKN2B*	42%	*CDKN2B*	32%	*CDKN2B*	33%			*TMRPSS2-ERG*	16%	*TMRPSS2-ERG*	16%
	*ASXL1*	14%	*KDM6A*	42%	*ARID1A*	26%	*ARID1A*	25%			*CDKN2A*	13%	*CDKN2A*	13%
	*BAP1*	14%	*PIK3CA*	29%	*MLL2*	22%	*MLL2*	21%			*MYC*	12%	*MYC*	12%
	*CCND2*	14%	*STAG2*	25%	*PIK3CA*	23%	*PIK3CA*	24%			*PIK3CA*	9%	*PIK3CA*	9%
	*CDK6*	14%	*ARID1A*	21%	*KDM6A*	22%	*KDM6A*	24%			*ATM*	8%	*ATM*	8%
	*KDM5A*	14%	*CREBBP*	18%	*STAG2*	17%	*STAG2*	18%			*CDKN2B*	8%	*CDKN2B*	8%
	*KDM5C*	14%	*MLL2*	13%	*ERBB2*	14%	*ERBB2*	13%			*CDK12*	7%	*CDK12*	7%
MSI High Status	0% (0/6)		0% (0/15)		0% (0/136)		0% (0/151)		0% (0/1)		3% (4/133)		3% (4/134)	
Median TMB	3.6		7.8		6.25		7		6.3		3.5		3.5	
TMB >10 mut/Mb	0%		25%		30%		30%		0%		5.7%		6%	
TMB >20 mut/Mb	0%		8.0%		11%		10%		0%		4.50%		4%	
PD-L1 Low	0% (0/2)		100% (1/1)		20% (3/15)		25% (4/16)		0% (0/1)		11% (4/35)		11% (4/35)	
PD-L1 High	0% (0/2)		0% (0/1)		13% (2/15)		13% (2/16)		0% (0/1)		0% (0/35)		0% (0/36)	

Within UBC, co-mutations in TERT showed statistically significant reduction, while co-mutations in KDM6A demonstrated considerable increase (both *p* < 0.05) when comparing cases harboring KRAS G12C genomic alterations versus KRAS G12C-negative tumors (Table 1). TP53, CDKN2A, TERT, CDKN2B, and KDM6A alterations represented the most prevalent concurrent genomic changes in the 24 UBC specimens with KRAS G12C mutations (Table 1 and Figure 1). PD-L1 demonstrated expression as low-positive tumor cell in the single KRAS G12C genomic alteration specimen (1/24) that underwent PD-L1 assessment.

**Figure 1:**
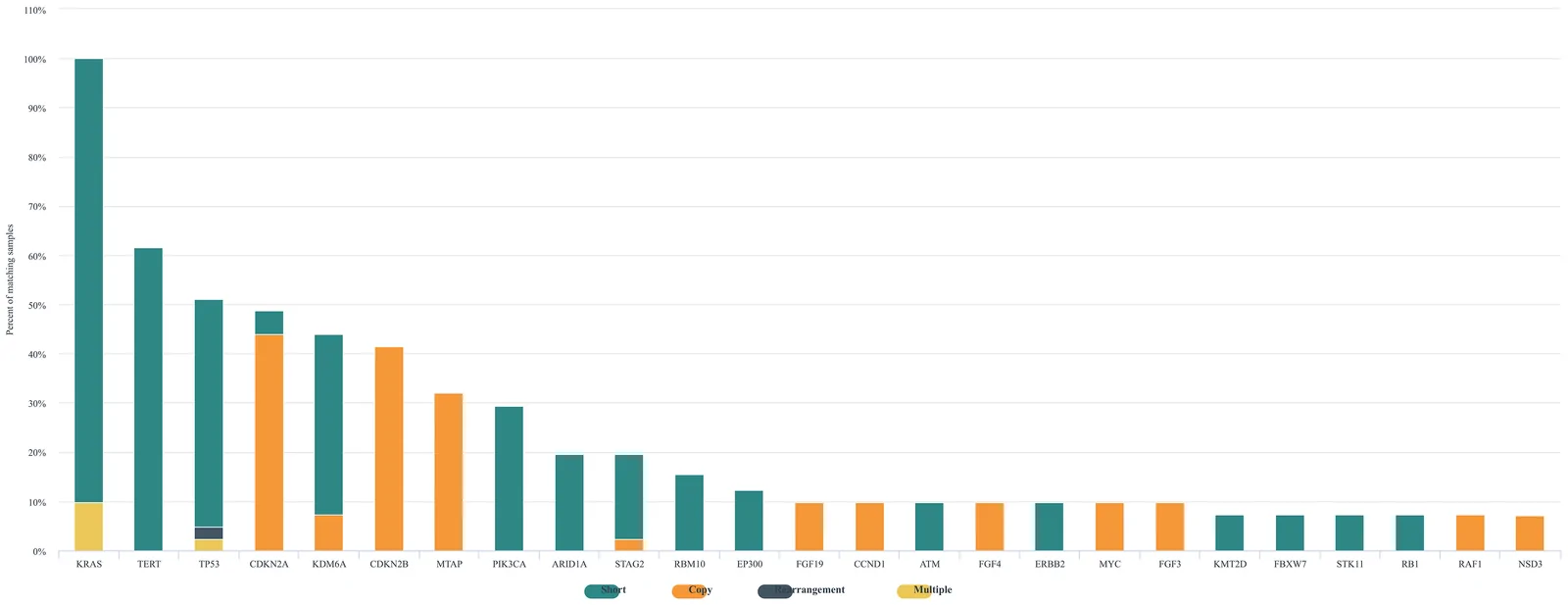
Distribution of mutations in KRAS mutated cases of urothelial bladder carcinoma.

In the PAAC specimen harboring a KRAS G12C genomic alteration, STK11 and APC genomic changes were co-mutated (Table 1). PD-L1 was not expressed in this particular specimen, though this case demonstrated elevated median TMB relative to other KRAS-mutated specimens (Figure 2).

**Figure 2:**
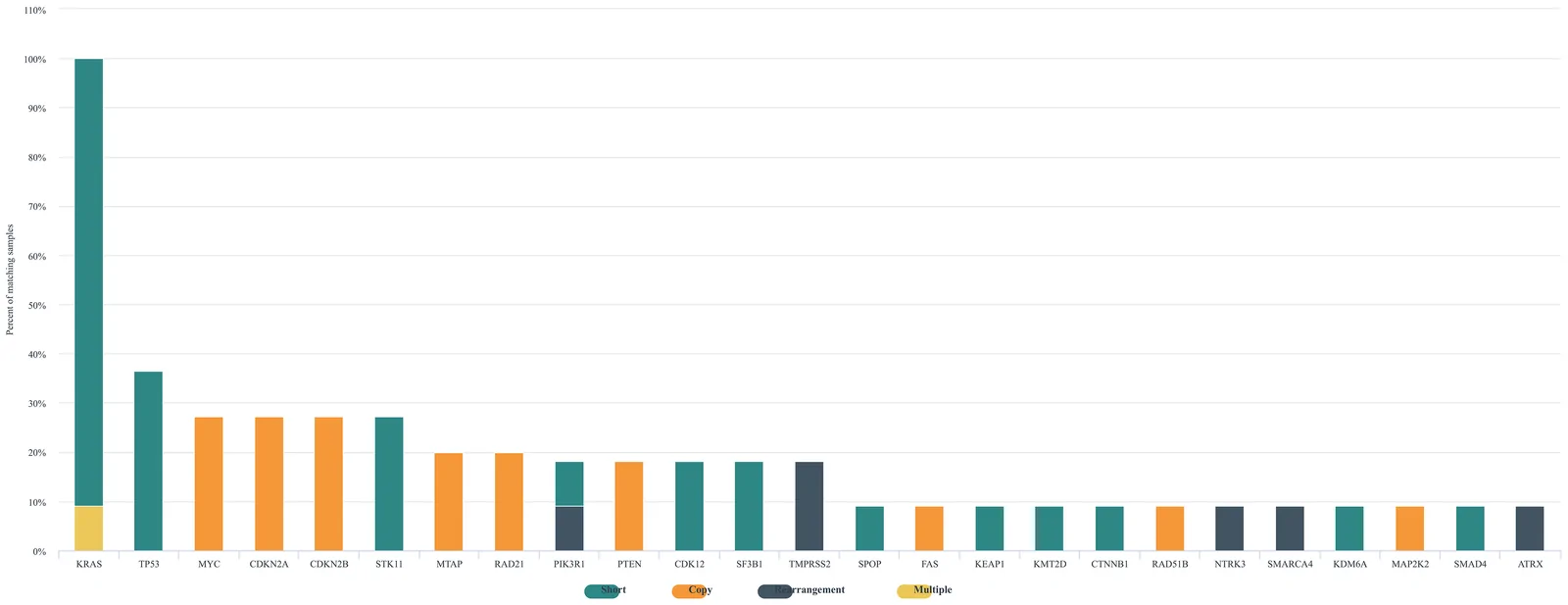
Distribution of mutations in KRAS mutated cases of prostate acinar adenocarcinoma.

## DISCUSSION

The preponderance of research investigating KRAS G12C alterations has concentrated on NSCLC, CRC, and pancreatic malignancies, as these neoplasms demonstrate this mutation with greater frequency. The clinical significance of KRAS G12C alterations in oncology has become increasingly prominent following the regulatory approval and FDA authorization of agents specifically targeting this alteration. Sotorasib, the pioneering FDA-authorized KRAS G12C inhibitor, demonstrated 36% objective response rate in targeted populations, requiring 10 months for this outcome, and has been utilized as second-line therapy in NSCLC [7].

Given the findings presented above, the distribution of KRAS alterations was characterized, with particular emphasis on KRAS G12C variants in genitourinary malignancies, to evaluate whether KRAS G12C mutations could represent a viable therapeutic target in genitourinary cancers.

Upon comparing other genetic aberration frequencies identified in genitourinary malignancies with KRAS G12C, this investigation demonstrated that KDM6A exhibited considerable elevation while TERT showed substantial reduction in KRAS G12C-mutated UBC when stratified against KRAS G12C-negative tumors. Additionally, STK11 and APC alterations were identified alongside the sole patient with KRAS G12C genomic alteration in PAAC, though these did not constitute co-mutations in non-KRAS G12C genomic alteration disease. This observation may carry implications for the therapeutic efficacy of different treatment approaches targeting specific alterations and warrants additional investigation.

This investigation represents one of limited studies comparing other clinical and genomic characteristics, including age, tumor mutational burden (TMB), and programmed death ligand 1 (PD-L1) expression, in genitourinary malignancies specifically. The paucity of research on KRAS G12C alterations and their associated co-mutations in genitourinary malignancies constrained the ability to contextualize this data relative to prior investigations, emphasizing the value of this work in contributing to a more comprehensive understanding of this alteration.

Lee and colleagues reported that in a pan-tumor evaluation, patients harboring KRAS G12C alterations demonstrated older age, increased female representation, elevated TMB, and greater likelihood of high PD-L1 expression [2]. Direct comparison to this study’s findings proves challenging given the inclusion of a broader malignancy spectrum; however, the results can be compared to the general trend of the alteration. This investigation identified that patients with KRAS G12C mutations in UBC demonstrated marginally elevated median age. Also, the patients with the mutation in UBC and PAAC exhibited elevated median TMB relative to the cohort from Lee and colleagues [2]. Nevertheless, this study demonstrated that the predominant proportion of affected patients were male, and the mutation showed reduced PD-L1 expression if any expression occurred. Comparative limitations to pan-cancer analyses exist and the above comparison should be interpreted with care. The PAAC results demonstrated only one KRAS G12C positive case, therefore constraining the available data for comparison. Additionally, only 1 UBC specimen out of 24 KRAS G12C genomic alteration-positive specimens underwent PD-L1 assessment.

## MATERIALS AND METHODS

Institutional approval for this study, including a waiver of informed consent requirements, was secured from the Western Institutional Review Board (Protocol No. 20152817). Tissue specimens from 1,453 renal clear cell carcinomas (RCCC), 3,879 urothelial bladder carcinomas (UBC), and 8,322 prostate acinar adenocarcinomas (PAAC) underwent comprehensive genomic profiling (CGP) using hybridization-captured, adaptor ligation-based DNA libraries. All specimens were from patients with metastatic disease at the time of nucleic acid extraction. Patient characteristics including age and sex, standard histopathologic features, immunohistochemical staining profiles, and tissue source confirmation for the cases submitted were retrieved from clinical documentation and pathology assessment records. One hematoxylin and eosin-stained tissue section corresponding to the specimen submitted for genomic analysis was available per case for morphologic characteristic review (Supplementary Figure 1). All specimens processed for DNA isolation contained a minimum of 20% neoplastic nuclei. Specimens underwent evaluation using comprehensive coding exons from at least 324 cancer-associated genes, plus select intron sequences from at least 31 genes frequently rearranged in cancer, as previously described by Milbury and colleagues [8]. The bioinformatics processing methodology employed in this study utilized Bayesian algorithms to detect base substitutions, local assembly algorithms to identify short insertions and deletions, comparison with process-matched normal control samples to determine gene copy number alterations, and chimeric read pair analysis to identify gene fusions, as previously outlined by Frampton et al. [9]. Visualization of sequencing data results was achieved using OncoPrint plot generation through the cBio portal as described by Gao et al. [10] and Cerami et al. [11]. Microsatellite instability assessment was accomplished by DNA sequencing of at least 95 loci, as previously documented [12]. For microsatellite loci demonstrating repeat length of 7 base pairs or greater, next-generation sequencing-based microsatellite instability scoring was performed, with categorical stratification into microsatellite instability-high, microsatellite instability-intermediate, or microsatellite stable designations using unsupervised hierarchical clustering of specimens based on microsatellite instability status, as previously evaluated via gold standard methodologies [13]. PD-L1 expression was determined on tumor tissue subsets using the DAKO 22C3 assay, with low-positive tumor cell scoring defined as 1–49% staining and high-positive tumor cell scoring defined as ≥50% staining. Fisher's exact test and Mann-Whitney *U*-test were employed for statistical comparisons between the two cohorts. Statistical significance was established at *p* < 0.05.

## CONCLUSIONS

This investigation demonstrates that KRAS G12C alterations occur rarely in major genitourinary malignancies. Despite this, the growing therapeutic relevance and potential for enrollment in basket-type clinical trials utilizing novel anti-KRAS G12C therapeutic agents warrants additional study of these alterations in genitourinary malignancies. This holds particular significance for bladder and prostatic carcinomas. While these novel therapeutic approaches may not prove beneficial for the majority of patients with these malignancies, they may become integral in managing the subset of patients harboring the KRAS G12C alteration.

## SUPPLEMENTARY MATERIALS



## Abbreviations

RCCC: Renal Clear Cell Carcinoma
UBC: Urothelial Bladder Carcinoma
PAAC: Prostate Acinar Adenocarcinoma
NSCLC: Non-Small Cell Lung Cancer
CRC: Colorectal Adenocarcinoma
GA: Genomic Alteration
CGP: Comprehensive Genomic Profiling
TMB: Tumor Mutational Burden
FFPE: Formalin-fixed paraffin-embedded

## References

[1] Tate JG, Bamford S, Jubb HC, Sondka Z, Beare DM, Bindal N, Boutselakis H, Cole CG, Creatore C, Dawson E, Fish P, Harsha B, Hathaway C, et al. COSMIC: the Catalogue Of Somatic Mutations In Cancer. Nucleic Acids Res. 2019; 47:D941–47. 10.1093/nar/gky1015. 30371878 PMC6323903

[2] Lee JK, Sivakumar S, Schrock AB, Madison R, Fabrizio D, Gjoerup O, Ross JS, Frampton GM, Napalkov P, Montesion M, Schutzman JL, Ye X, Hegde PS, et al. Comprehensive pan-cancer genomic landscape of KRAS altered cancers and real-world outcomes in solid tumors. NPJ Precis Oncol. 2022; 6:91. 10.1038/s41698-022-00334-z. 36494601 PMC9734185

[3] Kwan AK, Piazza GA, Keeton AB, Leite CA. The path to the clinic: a comprehensive review on direct KRASG12C inhibitors. J Exp Clin Cancer Res. 2022; 41:27. 10.1186/s13046-021-02225-w. 35045886 PMC8767686

[4] Prior IA, Hood FE, Hartley JL. The Frequency of Ras Mutations in Cancer. Cancer Res. 2020; 80:2969–74. 10.1158/0008-5472.CAN-19-3682. 32209560 PMC7367715

[5] Nassar AH, Adib E, Kwiatkowski DJ. Distribution of KRASG12C Somatic Mutations across Race, Sex, and Cancer Type. N Engl J Med. 2021; 384:185–87. 10.1056/NEJMc2030638. 33497555

[6] AACR Project GENIE Consortium. AACR Project GENIE: Powering Precision Medicine through an International Consortium. Cancer Discov. 2017; 7:818–31. 10.1158/2159-8290.CD-17-0151. 28572459 PMC5611790

[7] Skoulidis F, Li BT, Dy GK, Price TJ, Falchook GS, Wolf J, Italiano A, Schuler M, Borghaei H, Barlesi F, Kato T, Curioni-Fontecedro A, Sacher A, et al. Sotorasib for Lung Cancers with *KRAS* p.G12C Mutation. N Engl J Med. 2021; 384:2371–81. 10.1056/Nejmoa2103695. 34096690 PMC9116274

[8] Milbury CA, Creeden J, Yip WK, Smith DL, Pattani V, Maxwell K, Sawchyn B, Gjoerup O, Meng W, Skoletsky J, Concepcion AD, Tang Y, Bai X, et al. Clinical and analytical validation of FoundationOne®CDx, a comprehensive genomic profiling assay for solid tumors. PLoS One. 2022; 17:e0264138. 10.1371/JOURNAL.PONE.0264138. 35294956 PMC8926248

[9] Frampton GM, Fichtenholtz A, Otto GA, Wang K, Downing SR, He J, Schnall-Levin M, White J, Sanford EM, An P, Sun J, Juhn F, Brennan K, et al. Development and validation of a clinical cancer genomic profiling test based on massively parallel DNA sequencing. Nat Biotechnol. 2013; 31:1023–31. 10.1038/nbt.2696. 24142049 PMC5710001

[10] Gao J, Aksoy BA, Dogrusoz U, Dresdner G, Gross B, Sumer SO, Sun Y, Jacobsen A, Sinha R, Larsson E, Cerami E, Sander C, Schultz N. Integrative analysis of complex cancer genomics and clinical profiles using the cBioPortal. Sci Signal. 2013; 6:pl1. 10.1126/SCISIGNAL.2004088. 23550210 PMC4160307

[11] Cerami E, Gao J, Dogrusoz U, Gross BE, Sumer SO, Aksoy BA, Jacobsen A, Byrne CJ, Heuer ML, Larsson E, Antipin Y, Reva B, Goldberg AP, et al. The cBio cancer genomics portal: an open platform for exploring multidimensional cancer genomics data. Cancer Discov. 2012; 2:401–4. 10.1158/2159-8290.CD-12-0095. 22588877 PMC3956037

[12] Chalmers ZR, Connelly CF, Fabrizio D, Gay L, Ali SM, Ennis R, Schrock A, Campbell B, Shlien A, Chmielecki J, Huang F, He Y, Sun J, et al. Analysis of 100,000 human cancer genomes reveals the landscape of tumor mutational burden. Genome Med. 2017; 9:34. 10.1186/s13073-017-0424-2. 28420421 PMC5395719

[13] Trabucco SE, Gowen K, Maund SL, Sanford E, Fabrizio DA, Hall MJ, Yakirevich E, Gregg JP, Stephens PJ, Frampton GM, Hegde PS, Miller VA, Ross JS, et al. A Novel Next-Generation Sequencing Approach to Detecting Microsatellite Instability and Pan-Tumor Characterization of 1000 Microsatellite Instability-High Cases in 67,000 Patient Samples. J Mol Diagn. 2019; 21:1053–66. 10.1016/J.JMOLDX.2019.06.011. 31445211 PMC7807551

