# Supplementary material for: Incidence of KRAS G12C mutations in genitourinary malignancies; emerging target in precision medicine
Source: Oncotarget. 2026 Aug 19;17:381–7. doi: 10.18632/oncotarget.28912 (PMC13637952; doi:10.18632/oncotarget.28912)
Supplement: Supplementary file 1 [file oncotarget-17-28912-s001.pdf]

Incidence of *KRAS* G12C mutations in genitourinary malignancies; emerging target in precision medicine

SUPPLEMENTARY MATERIALS

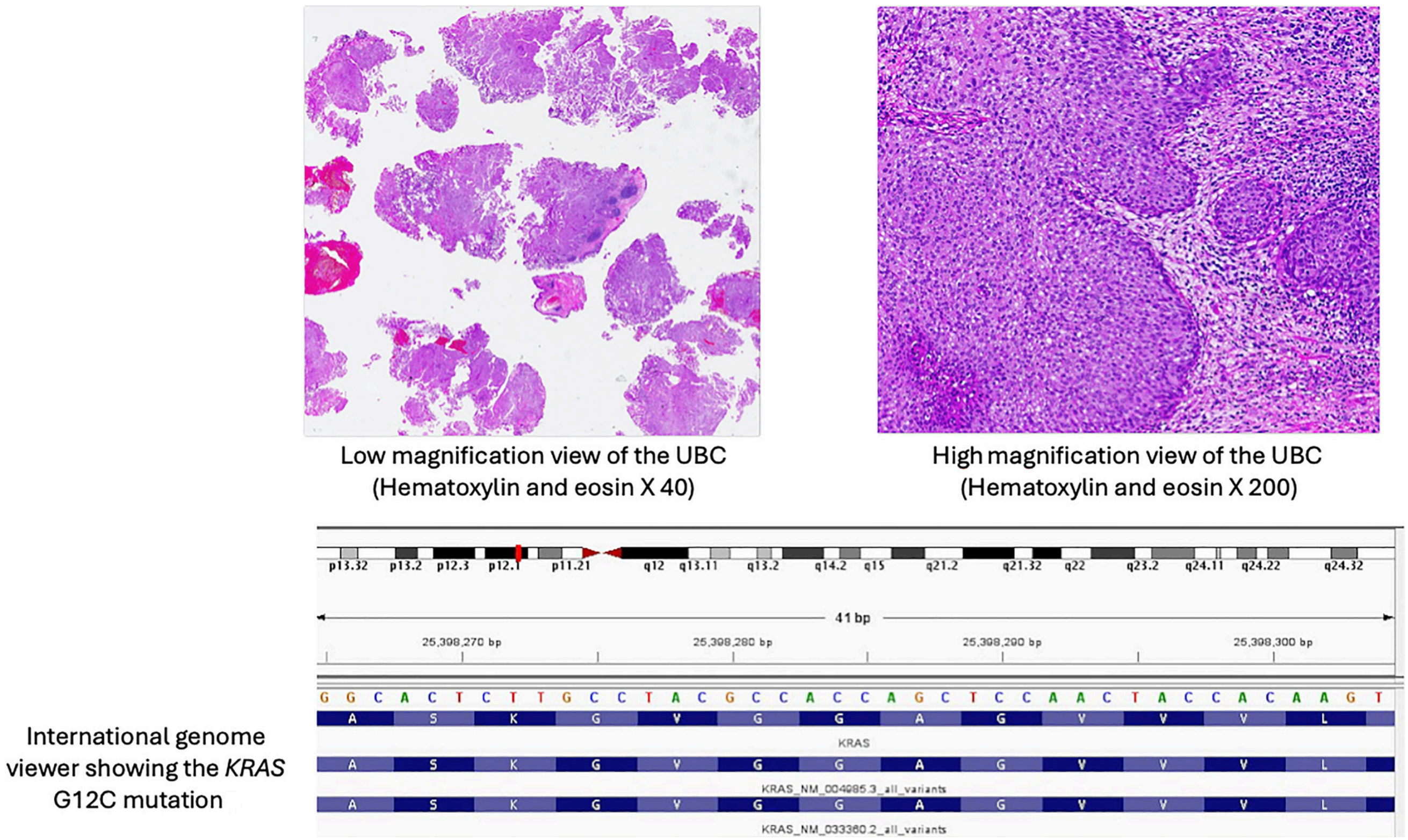

Supplementary Figure 1: TURBT of a high-grade urothelial bladder carcinoma that progressed to advanced disease in a 67-year-old man. On comprehensive genomic profiling, this tumor was MS stable and had a low tumor mutation burden of 4 mutations/Mb. In addition to a KRAS G12C missense mutation (IGV image), the copy number plot showed homozygous loss of CDKN2AIB and MTAP along with additional short variant mutations in PTEN Q17* and TERT promoter -124C> T.
